# Cloning, Synthesis and Functional Characterization of a Novel α-Conotoxin Lt1.3

**DOI:** 10.3390/md16040112

**Published:** 2018-03-31

**Authors:** Jinqin Chen, Li Liang, Huying Ning, Fengtao Cai, Zhuguo Liu, Longxiao Zhang, Liangyi Zhou, Qiuyun Dai

**Affiliations:** 1 Beijing Institute of Biotechnology, Beijing 100071, China; chenjq0210@163.com (J.C.); ranran21501@163.com (L.L.); tmu_ninghy@163.com (H.N.); fengtaoc@126.com (F.C.); liuzhuguo@126.com (Z.L.); chonglai1988@163.com (L.Zhang); m13517221660@163.com (L.Zhou); 2 Institute of Physical Science and Information Technology, Anhui University, Hefei 236041, China

**Keywords:** α-conotoxins, Lt1.3, cloning, synthesis, α3β2 nAChRs, GABA_B_R-coupled Cav2.2

## Abstract

α-Conotoxins (α-CTxs) are small peptides composed of 11 to 20 amino acid residues with two disulfide bridges. Most of them potently and selectively target nicotinic acetylcholine receptor (nAChR) subtypes, and a few were found to inhibit the GABA_B_ receptor (GABA_B_R)-coupled N-type calcium channels (Cav2.2). However, in all of α-CTxs targeting both receptors, the disulfide connectivity arrangement “C^1^-C^3^, C^2^-C^4^” is present. In this work, a novel α4/7-CTx named Lt1.3 (GCCSHPACSGNNPYFC-NH_2_) was cloned from the venom ducts of *Conus litteratus* (*C. litteratus*) in the South China Sea. Lt1.3 was then chemically synthesized and two isomers with disulfide bridges “C^1^-C^3^, C^2^-C^4^” and “C^1^-C^4^, C^2^-C^3^” were found and functionally characterized. Electrophysiological experiments showed that Lt1.3 containing the common disulfide bridges “C^1^-C^3^, C^2^-C^4^” potently and selectively inhibited α3β2 nAChRs and not GABA_B_R-coupled Cav2.2. Surprisingly, but the isomer with the disulfide bridges “C^1^-C^4^, C^2^-C^3^” showed exactly the opposite inhibitory activity, inhibiting only GABA_B_R-coupled Cav2.2 and not α3β2 nAChRs. These findings expand the knowledge of the targets and selectivity of α-CTxs and provide a new structural motif to inhibit the GABA_B_R-coupled Cav2.2.

## 1. Introduction

Conotoxins (CTx) are small, disulfide-rich peptides secreted by venom salivary glands of marine cone snails, whose precursors are encoded by various gene superfamilies [1,2]. Based on the consensus signal sequences of precursors, the number of cysteine residues and the arrangement of the disulfide bonds, CTx are categorized into various super-families (A, B, C, D, E, I, M, O, P, S, T, etc.) [1,2]. To date, more than 28 super-families have been identified [2]. Some conotoxins potently and selectively target a wide variety of ion channels, including sodium (Na^+^)-, potassium (K^+^)-, and calcium (Ca^2+^)-channels [3,4,5]. Certain conotoxins can also inhibit membrane receptors, including nicotinic acetylcholine receptor (nAChR) [6], 5-hydroxytryptamine receptor (5-HT3R) [7], *N*-methyl-d-aspartate receptors (NMDAR) [8], G-protein-coupled receptors (GPCRs) [9] and γ-aminobutyric acid type A receptor (GABA_A_R) [10]. They are highly valuable for neuropharmacological probes and drug development leads [11].

Most of α-CTxs belong to the A-superfamily of CTxs and selectively inhibit nAChRs. Their sizes range from 12 to 20 amino acid residues with two disulfide bonds [2,12]. According to the residue numbers of the inter cysteine loops (-CC-(loop1)-C-(loop2)-C-), they can be further divided into several subfamilies, such as α3/5, α4/3, α4/4, α4/6, α4/7, and α5/5 [2,12,13]. Among all α-CTxs, α4/7 CTxs are the most common, and have high potentials for development of selective inhibitors of neuronal nAChRs or N-type calcium ion channels [12,14], making these conotoxins valuable for developing neuropharmacological probes and drug leads of neuropathic pain and Alzheimer’s disease [15,16]. A few α4/7 CTxs, such as Vc1.1 [17], PeIA [18], AuIB [18], and Vc1.2 [19], were also found to inhibit the GABA_B_R-coupled N-type calcium channels (Cav2.2), which has now become a new target for developing pain-relief drugs. Vc1.1 also displays potent analgesic activity in rat partial sciatic nerve injury (PNL) and the chronic constriction injury model (CCI) [20].

This article describes the cloning, synthesis, and functional characterization of a novel α4/7 CTx named Lt1.3 (GCCSHPACSGNNPYFC-NH_2_), which was from the worm-hunting cone snail, *Conus litteratus* (*C. litteratus*), using the conserved untranslated region and the intron of A-superfamily conotoxins [21]. Two Lt1.3 isomers, Lt1.3-I and Lt1.3-II, with the respective disulfide bridges “C^1^-C^4^, C^2^-C^3^” and “C^1^-C^3^, C^2^-C^4^”, were then chemically synthesized and functionally characterized. Inhibition of nAChRs expressed in *Xenopus* oocytes by Lt1.3 was determined using two-electrode voltage-clamp. The results showed that Lt1.3-II (C^1^-C^3^, C^2^-C^4^) potently and selectively inhibited α3β2 nAChRs, but the isomer Lt1.3-I (C^1^-C^4^, C^2^-C^3^) did not. In addition, the functional amino acids were also investigated for Lt1.3-II inhibition of α3β2 nAChRs. More importantly, Lt1.3-I potently inhibited the GABA_B_R-coupled Cav2.2 co-expressed in HEK293T cells, but Lt1.3-II did not. To the best of our knowledge, Lt1.3-I is first peptide with the disulfide bridges “C^1^-C^4^, C^2^-C^3^” that inhibits the GABA_B_R-coupled Cav2.2. This finding expands the knowledge of target and selectivity of α-CTxs and provides a new structural motif to inhibit GABA_B_R-coupled Cav2.2.

## 2. Results

### 2.1. Cloning of α-CTx Lt1.3

**Figure 1:**
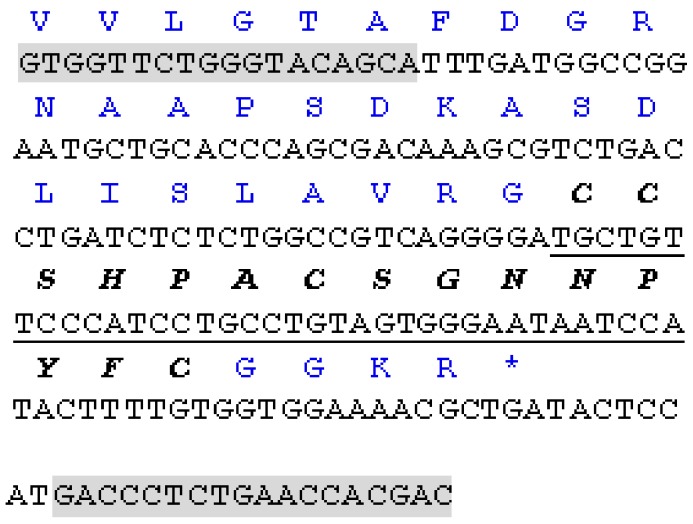
The partial cDNA sequence and predicted translation product of Lt1.3. The primer sequences are shaded. The codons of mature peptides are underlined. The nucleotide sequence data are available in the GenBank database under the accession numbers KF414121 for Lt1.3.

A novel α-CTx precursor was isolated from the genomic DNA of *C. litteratus* (Figure 1). The mature toxin sequence was predicted as GCCSHPACSGANPYFC-NH_2_ with a cysteine (C) pattern of CCX_4_CX_7_C (X represents any other amino acids). It belongs to cysteine framework I and the α4/7 CTx family, which is usually associated with an inhibitory activity for nAChRs. In accordance with conventional conotoxin nomenclature, the novel CTx was designated as Lt1.3 and its partial cDNA sequence of precursors has been submitted to GenBank and its genBank accession number is KF414121.

### 2.2. Peptide Synthesis and Disulfide Connectivity

**Figure 2:**
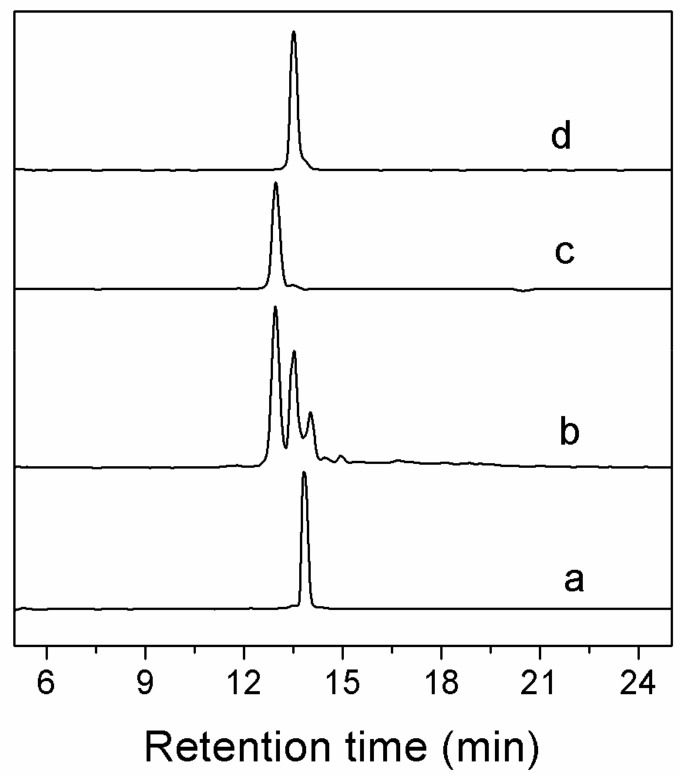
HPLC analyses of one-step folding products of linear Lt1.3. Traces from bottom to top: (**a**) the linear peptide; (**b**) one-step oxidized products; (**c**) the purified product of Lt1.3-I; and (**d**) the purified product of Lt1.3-II. Samples were applied to a Kromasil C18 column (5 μm, 4.6 mm × 250 mm) and eluted with a linear gradient of 5–10% B for 0–1 min; 10–50% B (B is acetonitrile containing 0.1% TFA) for 1–25 min. Absorbance was monitored at 214 nm. The flow rate was 1.0 mL/min.

**Figure 3:**
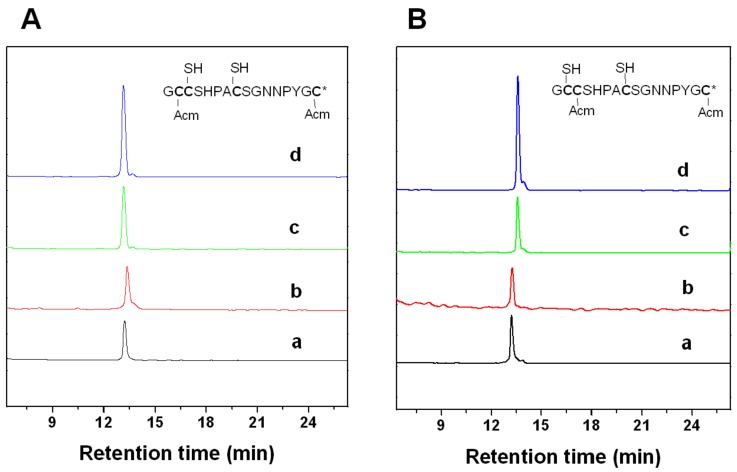
HPLC analyses of the folded products of linear Lt1.3 with Acm modification. Determination of the disulfide bond connectivity of Lt1.3-I (**A**) and Lt1.3-II (**B**). Traces from bottom to top: (**a**) linear peptide with Acm modifications at Cys1 and Cys3 or Cys 1 and Cys 4; (**b**) the first oxidized product; (**c**) the second oxidized product; and (**d**) the co-elution of the two-step folding products plus the purified product Lt1.3-I or Lt1.3-II (Figure 2). *: C-terminal caboxamide. Analytical conditions were the same as those described in Figure 2.

Two major peaks were found in the one-step folding products of Lt1.3 linear peptide analyzed by HPLC (Figure 2). According to the theoretical isomers of peptides with two disulfide bonds and the folding products, two Lt1.3 isomers (with disulfide connectivities “C^1^-C^3^, C^2^-C^4^” and “C^1^-C^4^, C^2^-C^3^”) were synthesized by the two-step oxidative folding strategy using the two different Acm-protected Lt1.3 linear peptides (Figure 3). Co-elution assays were performed with either Lt1.3-I (Figure 2c) or Lt1.3-II (Figure 2d) plus the two products formed in the two-step oxidative folding reaction (Figure 3). The results indicate that Lt1.3-II has a disulfide connectivity arrangement of “C^1^-C^3^, C^2^-C^4^”, and the Lt1.3-I has the arrangement of “C^1^-C^4^, C^2^-C^3^”. 

The Lt1.3 variants (Table 1) were also synthesized as described in the materials and methods section and assessed by analytical RP-HPLC. Their purities were >98% with the expected molecular weights.

### 2.3. Circular Dichroism Spectra of Lt1.3-II and Its Variants

**Figure 4:**
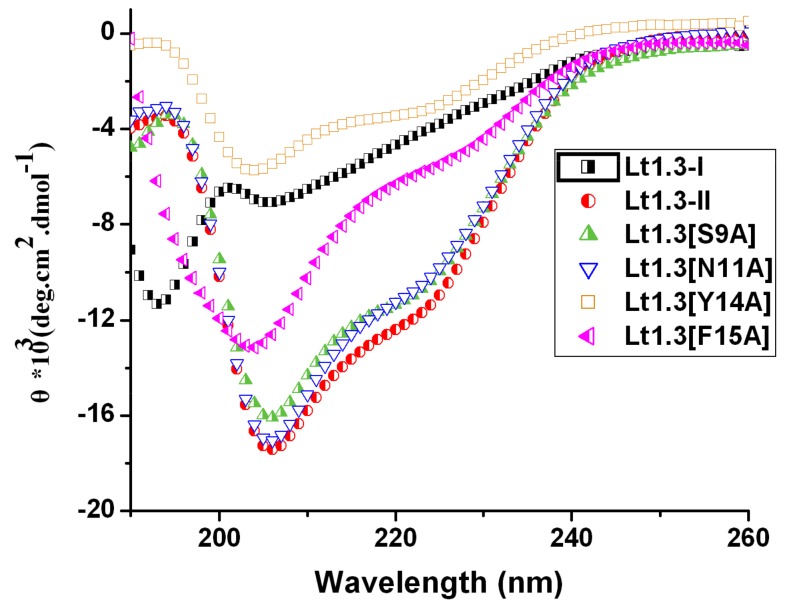
CD spectra of Lt1.3 in 0.01 M phosphate buffer solution (pH = 7.2, 50% TFE (2,2,2-trifluoroethanol).

The circular dichroism (CD) spectra of Lt1.3-II and its variants in 0.01 M phosphate buffer (50% TFE (2,2,2-trifluoroethanol)) showed some ellipticities around 208 nm and 220 nm (Figure 4) although the minimum values of several Lt1.3 variants were lower than that of Lt1.3. However, Lt1.3-I did not show the helical CD spectrum. These results are consistent with the previous reports that α4/7-CTxs have a short helical structure if they have the disulfide bridges “C^1^-C^3^, C^2^-C^4^” and a conserved proline residue exists in loop2 [22]. Thus, this structural feature was used to confirm the disulfide bridges of Lt1.3 variants. It should be noted that the minimum ellipticities of Lt1.3 and its variant in 0.01 M phosphate buffer (pH = 7.20) were at 194 nm and 208 nm (Figure S1), suggesting they may contain distorted α-helical structures.

### 2.4. Activities of Lt1.3 and Its Variants on nAChRs

**Figure 5:**
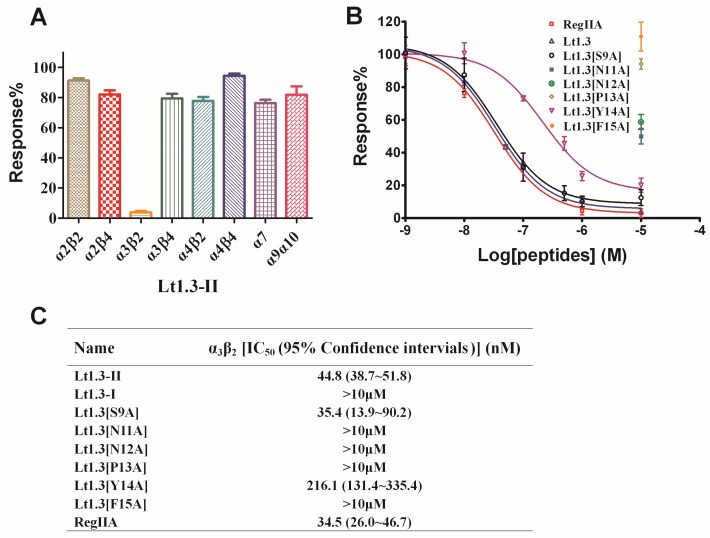
Effects of Lt1.3-II and variants on rat nAChRs expressed in *Xenopus* oocytes. (**A**) A bar graph of the mean ACH-evoked current amplitude of various rat nAChR subtypes in the presence of 10 μM Lt1.3-II (*n* = 3–4). (**B**) Concentration-dependent response curves of the rat α3β2 nAChRs (*n* = 4–6). (**C**) IC_50_ of peptides on various nAChR subtypes. The control peptide of α3β2 was RegIIA (IC_50_ = 34.5 (26.0–46.7) nM). IC_50_ of Lt1.3-II and its Ala variants for α3β2 was analyzed by GraphPad Prism and listed in Table 1. Data represent mean ± SEM.

Two-electrode voltage clamping was used to assess the effects of Lt1.3-II and Lt1.3-I on various neuronal nAChRs expressed by *Xenopus* oocytes. As shown in Figure 5, Lt1.3-II exhibited a strong inhibition on the rat neuronal subtype with an IC_50_ of 44.8 nM, but not on the subtypes of α2β2, α2β4, α3β4, α4β2, α4β4, α7 and α9α10 (Figure 5A, IC_50_ > 10 μM). On the other hand, Lt1.3-I displayed no apparent inhibitory effects on nAChR subtypes (IC_50_ > 10 μM) (Figure S2). These results indicate that Lt1.3-II selectively inhibits the α3β2 subtype of nAChRs, and its disulfide connectivity arrangement “C^1^-C^3^, C^2^-C^4^” is very important for the potency. When Ser^9^ was mutated to Ala, the inhibitory activity was similar. However, the replacement of Asn^11^, Asn^12^, Pro^13^, Tyr^14^, and Phe^15^ with Ala resulted in a sharp decrease in inhibitory activity on α3β2, the IC_50_ was decreased to 216 nM and <10 μM for Lt1.3[Y14A] and others (Figure 5B), respectively. 

### 2.5. Effects of Lt1.3 on the GABA_B_R-Mediated Cav2.2

**Figure 6:**
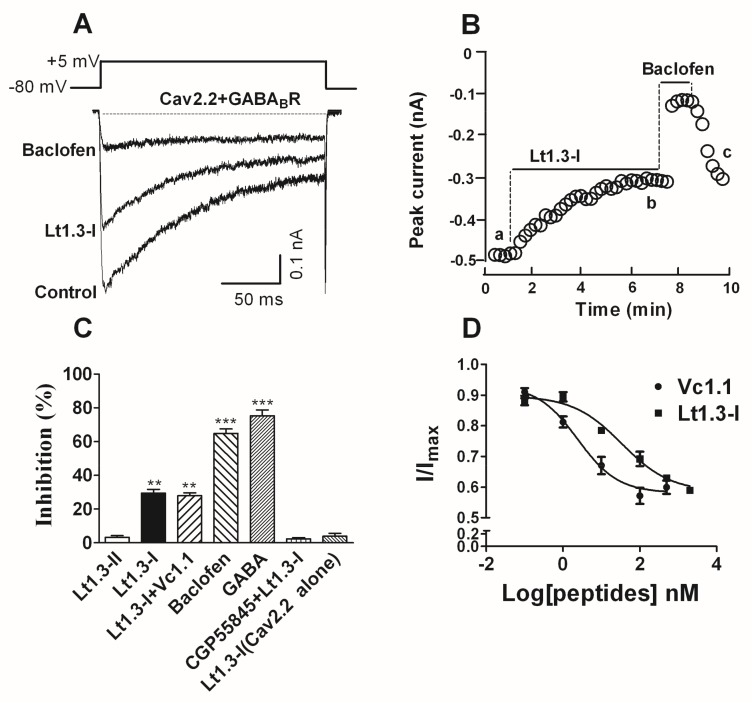
α-Conotoxin Lt1.3 inhibits Cav2.2 channels by activating GABA_B_R in HEK293T cells. (**A**) Representative superimposed current traces from HEK293T cells co-expressing human GABA_B_R and rat Cav2.2 channels in the absence and presence of Lt1.3-I (100 nM) and baclofen (10 μM, β-(4-chlorophenyl)-γ-aminobutyric acid). (**B**) Typical peak current amplitude plotted as a function of time in the inhibition of GABA_B_R-coupled Cav2.2 by Lt1.3-I (100 nM) and baclofen (10 μM). (**C**) Bar graph of inhibition of peak current amplitude by 10 μM Lt1.3-II (3.2 ± 1.8%), 100 nM Lt1.3-I (29.6 ± 4.9%), 100 nM Lt1.3-I + 10 nM Vc1.1 (28.0 ± 3.8%), 10 μM baclofen (64.9 ± 5.1%), 10 μM GABA (75.3 ± 6.7%), CPG55845 + 100 nM Lt1.3-I (2.3 ± 1.6%), 10 μM Lt1.3-I on Cav2.2 alone (4.0 ± 3.0%). Data represent the mean ± SEM. ** *p* < 0.01, *** *p* < 0.001 versus Lt1.3-II, one-way analysis of variance. (**D**) Concentration-response relationship for peptide inhibition of peak current in HEK293T cells co-expressing GABA_B_R and Cav2.2 channels. Data points represent averaged peak Ica amplitudes (I/Icontrol ± SEM); Values of IC_50_ Vc1.1 (2.4 nM (0.8–7.0)), Lt1.3-I (33.9 nM (10.7–107.1)) (*n* = 4–6 cells per data point).

The inhibitory activity of Lt1.3-I and Lt1.3-II to the GABA_B_R-coupled Cav2.2 co-expressed in HEK293T cells was determined. The results showed that Lt1.3-I displayed potent inhibitory activity towards the GABA_B_R-coupled Cav2.2 with the IC_50_ was 33.9 nM (Figure 6D), and Lt1.3-I lost activity in the presence of 1 μM CGP55845 (a GABA_B_R antagonist) (Figure 6C). On the contrary, Lt1.3-II did not inhibit the GABA_B_R-coupled Cav2.2 (IC_50_ > 10 μM, Figure 6C). In addition, Lt1.3-I did not directly inhibit Cav2.2 expressed in HEK293T cells (Figure 6C), suggesting an indirect mechanism for antagonism. 

## 3. Discussion

Currently, a dozen α4/7-CTXs have been found to target α3β2 and other nAChRs (Table 2). They share similar amino acid residues in the loop1 region, especially the conserved first (Ser) and third amino acid (Pro) [23]. The difference in selectivity is mainly derived from the surface-exposed charge and polarity of the loop2 region [24]. For example, the polarity, shape, and size of residues of α-CTX PnIA at position 10 affect the potency and selectivity to α3β2 and α7 nAChRs, while hydrophobic residues at position 10 maintain potency at both subtypes, smaller hydrophobic residues increase selectivity to α3β2 nAChRs [23]. Lt1.3-II has a small residue Gly at this position, so it selectively targets the α3β2 (IC_50_ = 44.8 nM) with high selectivity index (SI, >200) compared to other nAChR subtypes. 

The structure-activity relationship of Lt1.3 indicates that the substitution of Asn^11^, Asn^12^, Pro^13^, Tyr^14^, and Phe^15^ by Ala results in the sharp decrease in the inhibitory activity to α3β2 but the substitution of Ser^9^ (Figure 5) does not have this effect. The CD spectra show that Lt1.3[N11A] and Lt1.3 have similar α-helical structures (Figure 4), suggesting that Asn^11^ is a functional amino acid. However, the CD spectra of Lt1.3[N12A], Lt1.3[P13A], Lt1.3[Y14A], and Lt1.3[F15A] are significantly different from that of Lt1.3, indicating that the conformation play an important role in its potency. In addition, Tyr^14^ is unique to Lt1.3 since Glu, Asp, Asn, His and Gln commonly occur at this position in other 4/7-CTXs. Phe^15^ is also less common, although other hydrophobic residues, such as Leu, Ile, Val, and Tyr, are often present at this position. 

Two major peaks (Lt1.3-I and Lt1.3-II) existed during the oxidative folding reaction of the linear Lt1.3 peptides. Further activity assay demonstrated that Lt1.3-II (disulfide connectivity of “C^1^-C^3^ and C^2^-C^4^”) had a strong binding affinity to the α3β2 nAChRs, but Lt1.3-I, which has the disulfide connectivity of “C^1^-C^4^, C^2^-C^3^”, did not bind. This suggests that the globular and α-helical structure of Lt1.3-II is crucial for the potency to inhibit α3β2 nAChRs. 

To date, a number of α4/7 or α4/3 CTxs, such as Vc1.1 [17], PeIA [18], AuIB [19,34], Vc1.2 [19], and RgIA [35], have been found to inhibit α9α10 nAChRs and the GABA_B_R-coupled Cav2.2 (Cav2.2). They contain the disulfide bridges “C^1^-C^3^, C^2^-C^4^”. Surprisingly, Lt1.3-I with the disulfide bridges “C^1^-C^4^, C^2^-C^3^” exhibited potent inhibitory activity to the GABA_B_R-coupled Cav2.2, but Lt1.3-II with the disulfide bridges “C^1^-C^3^, C^2^-C^4^” did not. To the best of our knowledge, Lt1.3-I is first peptide with the disulfide bridges “C^1^-C^4^, C^2^-C^3^” that inhibits the GABA_B_R-coupled Cav2.2.

In conclusion, we found a novel α4/7 CTx Lt1.3 that targets specifically the α3β2 nAChR subtypes, and its isomer with the disulfide bridges “C^1^-C^4^, C^2^-C^3^” that specifically inhibits the GABA_B_R-coupled Cav2.2. This finding expands current knowledge of targets and selectivity of α-CTxs and provides a new structural motif to inhibit the GABA_B_R-coupled Cav2.2.

## 4. Materials and Methods

### 4.1. Cloning of Lt1.3 cDNA

Previous reports show that a long intron sequence exists between the exon I and exon II in the precursor gene sequence of α-conotoxins [21]. Exon I encodes the signal peptide and a part of the pro-region, while exon II encodes the other part of the pro-region, the mature peptide and 3′ untranslated region (3′-UTR). The sequences at the 5′ and 3′ end of introns are highly conserved and contains long dinucleotide (e.g., “GT”, “CA”) or trinucleotide (“CAT”) repeats. Taking advantage of the conserved 3′ end sequence of the intron, Lt1.3 was cloned from the genomic DNA of the venom ducts of *Conus litteratus* (*C. litteratus*) which was collected from the Cisha Island of the South China Sea, according to the kit protocol (TIANGEN catlog no: DP324, Beijing, China) [36]. The forward primer P1 (5′-GTGGTTCTGGGTCCAGCA-3′) from the conserved 3′ end sequence of intron identified in αA conotoxins and was paired with the reversed primer P2(5′-GTCGTGGTTCAGAUGGTC) from the conserved 3′-UTR sequences of A-family conotoxins described previously [14,36]. PCR amplification was carried out as follows: 94 °C, 4 min (1 cycle); 94 8 °C, 30 s; 55 8 °C, 30 s; 72 8 °C, 45 s (30 cycles); 72 8 °C, 10 min (1 cycle). The PCR products were analyzed by electrophoresis on agarose gel. The target band was excised from the gel and purified with Gel Extraction Mini Kit (Beyotime, Haimen, China). The purified PCR products were added to basyl A and ligated into the T-tailed plasmid vector pGEM-T for DNA sequencing. The predicted protein sequences were analyzed with software Seqtools (http://www.bio-soft.net/sms). The novel cDNA sequence Lt1.3 was identified following a comparison with the sequences available in the GenBank nucleotide sequence database and in the literature, and was deposited in the Genbank nucleotide sequence database.

### 4.2. Peptide Synthesis and Disulfide Connectivity Analysis

Lt1.3 and its variants were synthesized using the method described previously [18,37]. Briefly, Lt1.3 or its mutant was assembled and then cleaved from Rink resin. The released peptides (0.1 mg/mL) were oxidized in 0.1 M NH_4_HCO_3_ at room temperature, pH 8.0–8.2. The folding products were then purified by semi-preparative reverse phase-high performance liquid chromatography (RP-HPLC). The final products were assessed by analytical RP-HPLC. The primary sequences of Lt1.3 and its variants were listed in Table 1.

The disulfide connectivity of one-step oxidative folding products of Lt1.3 was analyzed by comparing the folded peptide products with known disulfide connectivity [38]. Briefly, linear peptides containing an acetamidomethyl (Acm)-protecting group at the C^2^-C^4^ or C^1^-C^4^ position were synthesized and then folded by incubation in 0.1 M NH_4_HCO_3_ (pH 8.0) at room temperature for 24–48 h. The folded products were further oxidized with an iodine mixture containing 30% CH_3_CN/2% TFA/68% H_2_O for 10 min to yield peptides with the -S-S- bridges of “C^1^-C^3^, C^2^-C^4^” or “C^1^-C^4^, C^2^-C^3^”. The mixture of this second oxidized product and the one-step folding product of Lt1.3 was analyzed by HPLC to determine the disulfide connectivity. The disulfide connectivity of Lt1.3 variants was determined according to its CD spectra. If they display typical α-helical circular dichroism (CD) spectra, the disulfide connectivity will be “C^1^-C^3^, C^2^-C^4^”.

### 4.3. Circular Dichroism (CD) Spectra 

CD spectra of Lt1.3 were measured between 190 and 340 nm on a Chirascan-plus Circular Dichroism spectrometers (Applied photophysics Ltd., Leatherhead, UK). The peptide was dissolved in 0.01 M phosphate buffer (pH 7.2) or 0.01 M PBS containing 50% TFE (2,2,2-trifluoroethanol) to a final concentration of 35 μM. A 1-mm path length quartz cell was employed. Each spectrum represented the accumulation of three individual scans collected at 1.0 nm intervals at a bandwidth of 1.0 nm.

### 4.4. Two-Electrode Voltage-Clamp Recording on Oocytes Expressing nAChRs

cDNA preparation, oocyte harvest and expression of nAChR subunits were performed as described previously [13,36]. Briefly, each *Xenopus* oocyte was injected with 30–40 ng of cRNA and incubated with ND96 solution containing 2.5 mM pyruvic acid sodium, 0.1 mg/mL BSA and antibiotics (10 U/mL penicillin, 10 μg/mL streptomycin) (Gibco by Life Technologies, Grand Island, NY, USA) at 18 °C. Electrophysiological experiments were performed at days 2–5 post-injection at room temperature (22 °C). The oocytes were gravity-perfused in a recording chamber (50 μL) with ND96 at a rate of 1.5 mL/min. The membrane potential was clamped at −70 mV and the ACh-gated currents were recorded with a two-electrode voltage-clamp amplifier Axoclamp 900A (Axon Instruments Inc., Union City, CA, USA). The perfusion medium was automatically switched among ND96, Ach (in ND96) and toxin (in ND96) using MPS-2 multichannel perfusion system (Inbio Life Science Instrument Co., Ltd., Wuhan, China). Ach, in 200 μM, 30 μM, and 100 μM amounts in ND96, was used to activate α7, α9α10, and other nAChRs subtypes, respectively. For the low-dose response, the oocyte was perfused with the toxin solution until equilibrated (5~10 min) and then activated by ACh. In high-dose experiments (1 μM or greater), 5.5 μL of a 10-fold concentrated toxin solution was directly pipetted into static bath 5 min prior to the exposure of ACh pulses.

The dose-dependent response data were fit to the equation: response (%) = 100/[1 + ([toxin]/IC_50_)*^n^*], where *n* is the Hill coefficient and IC_50_ is the inhibitor concentration giving half-maximal response, by non-linear regression analysis using GraphPad Prism 5 (GraphPad Software, San Diego, CA, USA).

### 4.5. HEK293T Cell Electrophysiology

HEK293T cells were transiently co-transfected with human GABAB1 and GABAB2 subunits (2 μg each, obtained from David J. Adams, University of Wollongong, Wollongong, Australia) and 0.2 μg mCherry fluorescent protein using Lipofectamine 2000 (Invitrogen, Van Allen Way, Carlsbad, CA, USA) according to the manufacturer’s protocol. After 24 h, the cells were then transiently co-transfected with rat Cav2.2 channels (α1B, β3, and α2δ1 subunits with 1 μg each) and 0.2 μg of the enhanced green fluorescent protein using Lipofectamine 2000 as well. Two to three days after transfection, cells were seeded on glass coverslips pretreated with poly-l-lysine and incubated at 37 °C in 5% CO_2_ for at least 6 h before recording. 

Whole-cell patch-clamp recording were performed as described previously [18,39]. Briefly, transfected HEK293T cells were superfused with a solution containing (in mM): NaCl 90, BaCl_2_ 10, CsCl 5, tetraethylammonium chloride (TEA-Cl) 30, MgCl_2_ 1, d-glucose 10, and HEPES 10, pH 7.4 with tetraethylammonium hydroxide (TEA-OH). Fire-polished borosilicate patch pipettes (2–3 MΩ tip resistance) were filled with a solution containing (in mM): K-gluconate 120, NaCl 5, MgCl_2_ 2, EGTA 5, MgATP 2, Na_2_GTP 0.6, and HEPES 10, pH 7.2 with CsOH. Whole-cell patch-clamp recordings were performed at room temperature (23–25 °C) using Multiclamp 700B amplifiers (Molecular Devices, Sunnyvale, CA, USA) controlled by Clampex 10.3/DigiData 1440A acquisition systems (Molecular Devices, Sunnyvale, CA, USA). Membrane currents were filtered at 2 kHz and sampled at 10 kHz. Leak and capacitive currents were subtracted using a 2P/4 pulse protocol. Peak current amplitude in response to the depolarizing pulse was measured once a steady state was achieved (3–5 min). All drugs were diluted to the appropriate final concentration and applied via perfusion. Baclofen (β-(4-chlorophenyl)-γ-aminobutyric acid) was used as a positive control for functional expression of GABA_B_R with Cav2.2 channels and the cell responding to baclofen with at least 50% peak current inhibition were included in our analysis. The dose-response data were fit to the equation: *Y* = *Y*min + (*Y*min − *Y*max)/(1 + 10 ^(Log IC_50_-*X*)^ × *h*), where *Y* is I/I_0_, *h* is the Hill coefficient (slope), and IC_50_ is the half-maximal inhibitory concentration. The non-linear regression analysis was performed using GraphPad Prism (GraphPad Software, San Diego, CA, USA).

## Figures and Tables

**Table 1:** Amino acid sequence of Lt1.3 and variants. *: C-terminal caboxamide.

Name	Amino Acid Sequences
Lt1.3	GCCSHPACSGNNPYFC *
Lt1.3[S9A]	GCCSHPACAGNNPYFC *
Lt1.3[N11A]	GCCSHPACSGANPYFC *
Lt1.3[N12A]	GCCSHPACSGNAPYFC *
Lt1.3[P13A]	GCCSHPACSGNNAYFC *
Lt1.3[Y14A]	GCCSHPACSGNNPAFC *
Lt1.3[F15A]	GCCSHPACSGNNPYAC *
RegIIA	GCCSHPACNVNNPHIC *

**Table 2:** Amino acid sequences and selectivity of α4/7-CTxs targeting nAChR α3β2. ^a^: Conserved amino acid are denoted by light gray shade; the scaffolds formed by disulfide-bonded cysteines are in boldface and boxed; ^b^: all the targets are rat nAChRs unless otherwise indicated; h: indicates human nAChRs; *: C-terminal carboxamide; γ: γ-carboxyglutamate; O: 4-trans-hydroxyproline; sTy: sulfated tyrosine.

α-CTX	Amino Acid Sequence ^a^	IC_50_ (nM) ^b^	Reference
Lt1.3	GCCSHPACSGNNPYFC *	α3β2 (44.8)	This work
AnIB	GGCCSHPACAANNQD(sTy)C *	α3β2 (0.3), α7 (76)	[25]
ArIA	IRDECCSNPACRVNNOHVCRRR	α3β2 (18.0), α7 (6.0)	[26]
ArIB	DECCSNPACRVNNPHVCRRR	α3β2 (60.1), α7 (1.8)	[26]
GIC	GCCSHPACAGNNQHIC *	hα3β2 (1.1), hα4β2 (309), hα3β4 (755)	[27]
GID	IRDγCCSNPACRVNNOHVC	α3β2 (3.1), α4β2 (152), α7 (4.5)	[28]
LsIA	SGCCSNPACRVNNPNIC *	α3β2 (10.3), α7 (10.1)	[29]
LvIA	GCCSHPACNVSHPEI C *	α3β2 (8.7), α6α3β2β3 (108)	[30]
MII	GCCSNPVCHLEHSNLC *	α3β2 (0.5), α7 (~200), α4β2 (548)	[31]
Mr1.7	PECCTHPACHVSHPELC *	α3β2 (53.1), α9α10 (187.5)	[12]
OmIA	GCCSHPACNVNNPHICG *	α3β2 (11.0), α7 (27.1)	[32]
PeIA	GCCSHPACSVNHPEL C *	α3β2 (23), α9α10 (6.9), α3β4 (480), α7 (1800)	[33]
PnIA	GCCSLPP CAANNPDYC *	α3β2 (9.6), α7 (252)	[31]
RegIIA	GCCSHPACNVNNPHIC *	α3β2 (33), α3β4 (97), α7 (103), α9α10 (>1000)	[26]
TxIA	GCCSRPP CIANNPDL C *	α3β2 (3.6), α7 (392)	[30]
Vc1.1	GCCSDPRCNYDHPEI C *	α3β2 (5532), α9α10 (109), α3β4 (4200), α7 (7123)	[23]

## Supplementary Materials

The following are available online at http://www.mdpi.com/1660-3397/16/4/112/s1, Figure S1: CD spectra of Lt1.3 in 0.01 M phosphate buffer solution (pH = 7.2), Figure S2: Effects of Lt1.3-1 on rat nAChRs expressed in Xenopus Oocytes.
