# Supplementary material for: Cloning, Synthesis and Functional Characterization of a Novel α-Conotoxin Lt1.3
Source: Mar Drugs. 2018 Mar 31;16(4):112. doi: 10.3390/md16040112 (PMC5923399; doi:10.3390/md16040112)
Supplement: Supplementary file 1 [file marinedrugs-16-00112-s001.pdf]

Supplementary Information

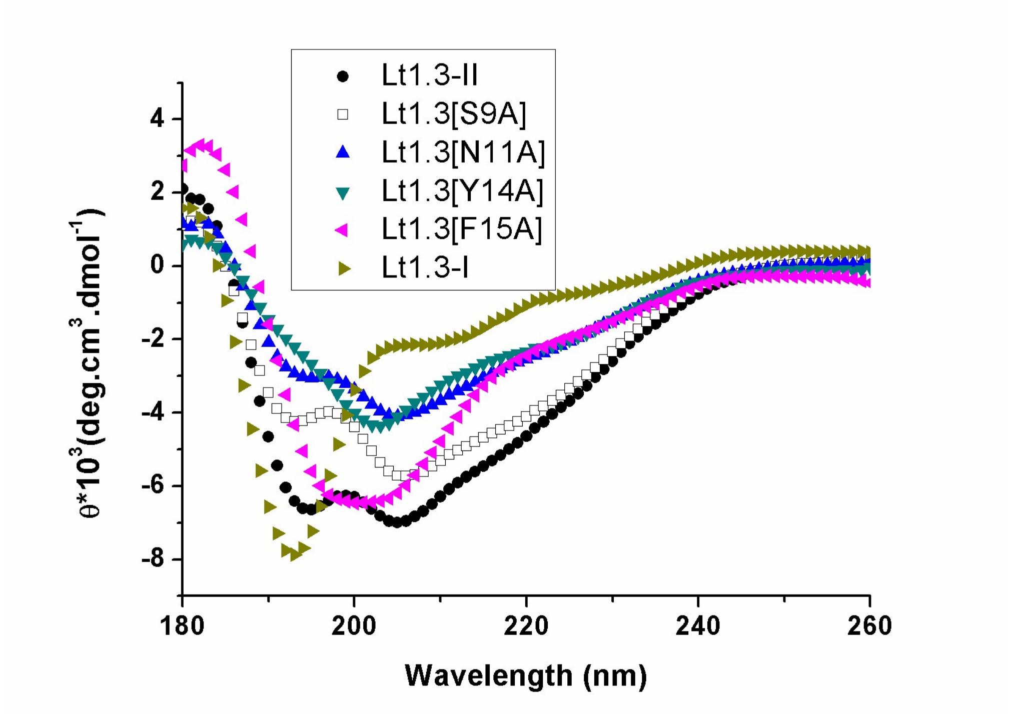

Figure S1. CD spectra of Lt1.3 in 0.01 M phosphate buffer solution (pH=7.2)

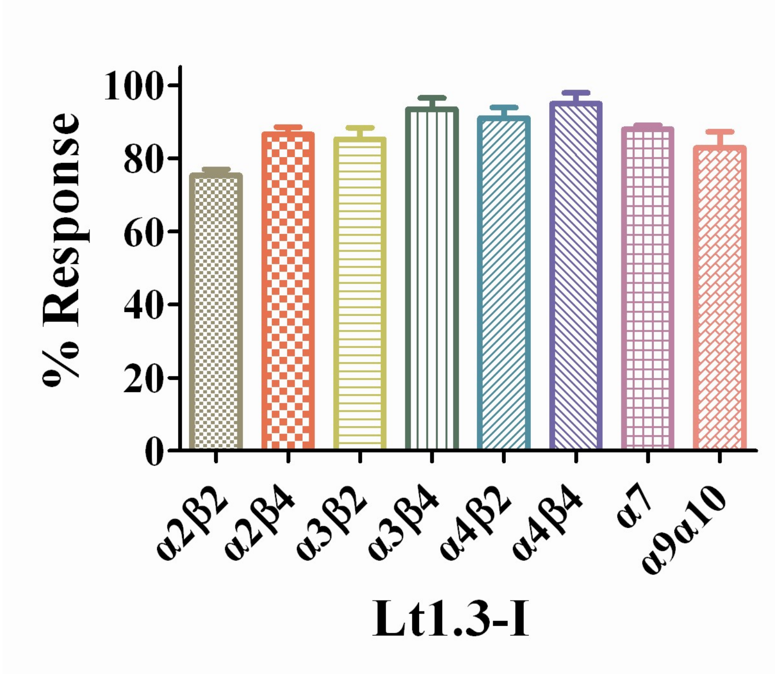

Figure S2. Effects of Lt1.3-1 on rat nAChRs expressed in *Xenopus Oocytes*. **The i**nhibition ration of 10 μM Lt1.3-I for various rat nAChR subtypes was < 50% (n = 3-4). The data were expressed as mean ± SEM.
